# Kynurenines as a Novel Target for the Treatment of Malignancies

**DOI:** 10.3390/ph14070606

**Published:** 2021-06-23

**Authors:** Adrian Mor, Anna Tankiewicz-Kwedlo, Dariusz Pawlak

**Affiliations:** 1 Department of Pharmacodynamics, Medical University of Bialystok, Mickiewicza 2c, 15-222 Bialystok, Poland; dariusz.pawlak@umb.edu.pl; 2 Department of Monitored Pharmacotherapy, Medical University of Bialystok, Mickiewicza 2c, 15-222 Bialystok, Poland; anna.tankiewicz-kwedlo@umb.edu.pl

**Keywords:** tryptophan, kynurenine pathway, kynurenine, cancer, immune escape, indoleamine 2,3-dioxygenase, tryptophan 2,3-dioxygenase, enzyme inhibitors, cancer treatment

## Abstract

Malignancies are unquestionably a significant public health problem. Their effective treatment is still a big challenge for modern medicine. Tumors have developed a wide range of mechanisms to evade an immune and therapeutic response. As a result, there is an unmet clinical need for research on solutions aimed at overcoming this problem. An accumulation of tryptophan metabolites belonging to the kynurenine pathway can enhance neoplastic progression because it causes the suppression of immune system response against cancer cells. They are also involved in the development of the mechanisms responsible for the resistance to antitumor therapy. Kynurenine belongs to the most potent immunosuppressive metabolites of this pathway and has a significant impact on the development of malignancies. This fact prompted researchers to assess whether targeting the enzymes responsible for its synthesis could be an effective therapeutic strategy for various cancers. To date, numerous studies, both preclinical and clinical, have been conducted on this topic, especially regarding the inhibition of indoleamine 2,3-dioxygenase activity and their results can be considered noteworthy. This review gathers and systematizes the knowledge about the role of the kynurenine pathway in neoplastic progression and the findings regarding the usefulness of modulating its activity in anticancer therapy.

## 1. Introduction

**Figure 1:**
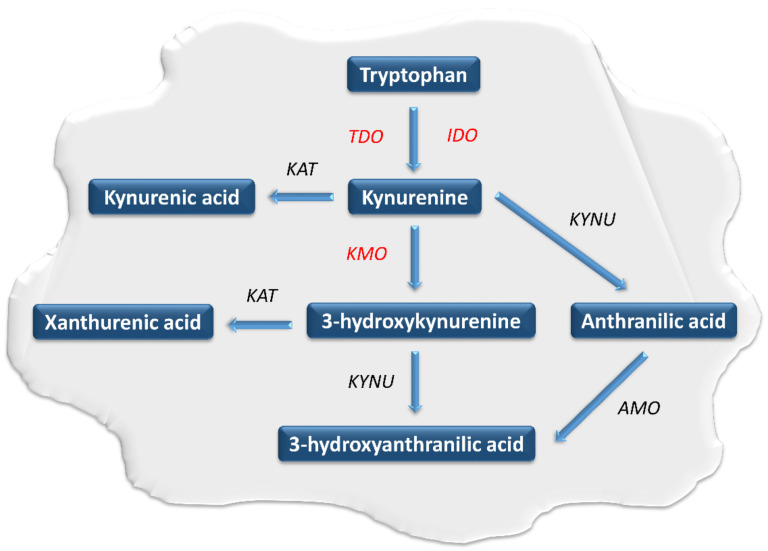
The course of the kynurenine pathway in neoplastic cells. The kynurenine pathway enzymes are in italics, while those involved in carcinogenesis are marked in red. AMO: aminocarboxymuconatesemialdehyde decarboxylase, IDO: indoleamine 2,3-dioxygenase, KAT: kynurenine aminotransferase, KMO: kynurenine 3-monooxygenase, KYN: kynurenine, KYNU: kynureninase, TDO: tryptophan 2,3-dioxygenase.

Tryptophan (TRP) belongs to the essential biogenic amino acids. It undergoes extensive metabolism through several metabolic pathways [1,2,3,4,5,6,7]. They play an important role in the regulation of numerous physiological and pathological processes. The kynurenine pathway (KP) is the main pathway responsible for the metabolism of this amino acid [2,3,4,5,6,7,8,9,10,11,12,13,14,15,16,17,18,19,20,21,22]. Within KP, TRP is oxidized into *N*-formylkynurenine using tryptophan 2,3-dioxygenase (TDO) and two indoleamine 2,3-dioxygenase isoforms (IDO-1 and IDO-2) [23,24]. Under physiological conditions, IDO and TDO catalyze the same reaction in parallel but have different tissue distributions. TDO activity is highest in the liver, while IDO occurs in almost all body tissues [25,26,27,28,29]. In the next step, *N*-formylokynurenine is converted to kynurenine (KYN) by the enzyme formamidase. KYN is metabolized by the three branches of KP, leading to the formation of kynurenic acid, anthranilic acid and 3-hydroxykynurenine (3-HKYN) [30,31,32,33]. The catabolism of KYN to 3-HKYN occurs with the participation of kynurenine 3-monooxygenase (KMO), while to kynurenic acid by kynurenine aminotransferase (KAT) [28,29]. In turn, 3-HKYN is converted to xanthurenic acid by the KAT and to 3-HAA after modification with the kynureninase (KYNU) (Figure 1) [34,35,36]. The above enzymes are important levels of the KP cascade because they participate in a synthesis of toxic metabolites of KP, responsible for the development of a wide range of systemic disorders, including neoplastic progression [2,3,4,5,6,7,8,9,10,11,12,13,14,15,16,17,18,19,20,21,22]. In 2005 Muller et al. were the first to evaluate the effectiveness of IDO inhibition in an in-vivo tumor model [37]. Research concerning this issue led the researchers to conclude that modulation of the KP activity has considerable potential as a new strategy for tumor treatment [37,38,39].

## 2. The Role of the Kynurenine Pathway in Neoplastic Processes

Effective cancer treatment is still a great challenge for modern medicine. Tumors have produced a broad range of mechanisms to evade an immune and therapeutic response [37,38,39,40]. An accumulation of the KP metabolites can enhance neoplastic progression because it suppresses immune processes directed against cancer cells. KYN is one of the most potent immunosuppressive metabolites belonging to this pathway. [40,41,42]. The expression and activity of IDO and TDO, responsible for its synthesis, are upregulated in most cancers. These enzymes are thought to be involved in the processes responsible for the development of immune escape of tumors and resistance to immuno-, radio- and chemotherapy [40,43,44,45,46,47].

**Figure 2:**
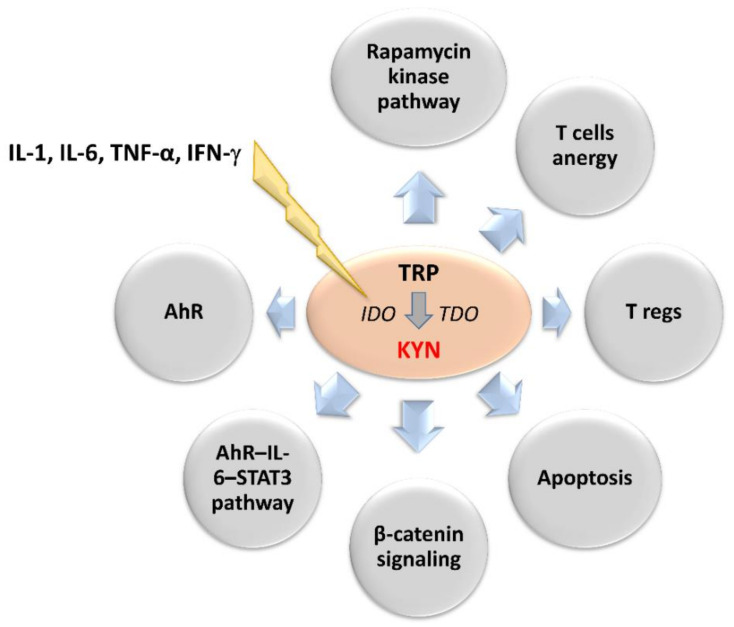
Upstream regulators of 2,3-dioxygenases activity and downstream effectors of kynurenine involved in neoplastic progression. IL-1, IL-6, TNF-α and IFN-γ stimulates IDO leading to enhanced synthesis of kynurenine. Its main effectors are presented in the circles. AhR: aryl hydrocarbon receptor, IDO: indoleamine 2,3-dioxygenase, IFN-γ: interferon-γ, IL-1: interleukin 1, IL-6: interleukin 6, KYN: kynurenine, STAT3: signal transducer and activator of transcription 3, TDO: tryptophan 2,3-dioxygenase, TNF-α: tumor necrosis factor-α, TRP: tryptophan.

IDO is activated in response to the release of pro-inflammatory factors such as interleukins (IL-1 and IL-6), tumor necrosis factor-α and interferon-γ (IFN-γ) [48,49,50]. It enhances immune tolerance by an increase in a KYN and its downstream metabolites levels within the tumor microenvironment [51,52]. KYN induces general control nonderepressible-2 kinase and mammalian target of rapamycin kinase pathways, which reduce the immune response [41,53,54,55,56]. Other effects of KYN include increased TH1 and effector T cells anergy and apoptosis rate and T regulatory cells (T regs) immunosuppressive activity [57,58,59,60]. In turn, IDO-activated T regs can promote IDO1 expression in dendritic cells (DCs) during the antigen presentation process, additionally enhancing the immunosuppressive mechanism [61,62]. The IDO-dependent KYN synthesis can also directly promote tumor growth and metastasis via the generation of pro-proliferative metabolites and activation of β-catenin signaling in IDO-expressing tumors [63,64]. Therefore, prolonged IDO1 overexpression in the tumor microenvironment negatively correlates with patient outcomes [46,65,66,67]. IDO-positive tumors and microenvironment tissues showed basal phosphorylation and acetylation of signal transducer and activator of transcription 3 (STAT3) [68,69]. The inhibition of IL-6 or STAT3 using small interfering RNA or pharmacological inhibitors decreases IDO activity. In turn, KYN activates the aryl hydrocarbon receptor (AhR), which upregulates the expression of IL-6 and STAT3. It indicates that IDO expression may be also upregulated by autocrine stimulation [18,19,20,70]. Inhibition of AhR signaling restores proliferation and activation of T cells in the IDO-expressing human cancers. This indicates IDO inhibitors and AhR antagonists as a new therapeutic solution in cancer therapy (Figure 2) [18,19,71,72].

The involvement of TDO in cancer biology seems to be similar to IDO. Its overexpression has been observed in several cancers [73,74,75]. As mentioned earlier, although IDO and TDO catalyze the same reaction, their tissue distribution is different [28,29,30]. Therefore, the impact of the TDO on the overall TRP metabolism cannot be ignored [73,74,75]. This is supported by the fact that isomers of 1-methyl-tryptophan (1-MT), commonly used in research as IDO inhibitors, do not fully inhibit TRP catabolism via KP. Although, preclinical studies found that targeting TDO significantly decreased the incidence of cancer progression and restored anticancer immunity [73,74,75,76], inhibition of IDO1 may enhance TDO expression and activity (Figure 2). Therefore, TDO inhibitors could be useful as auxiliary therapeutics. In summary, the inhibition of IDO, TDO, or both, impairing immune resistance of tumors, may represent a safe and efficient approach for cancer therapy, stimulating tumoral immune rejection and improving the efficacy of cancer immunotherapy [76]. 

## 3. Modulation of Kynurenine Pathway Activity in the Management of Neoplastic Diseases

### 3.1. Pharmacological Modulation of IDO and TDO Activity

Most approaches related to targeting KP involve pharmacological inhibition of enzymes belonging to this pathway. There is an increasing number of discovered IDO and TDO inhibitors [37,38,39,40,52,75,76,77]. Most of them are IDO1 inhibitors developed for cancer therapy [37,38,39,40,52]. Preclinical models and clinical trials showed that their administration in monotherapy slows the growth of tumors but did not eliminate or prevent them. However, their combination with chemotherapies or immunotherapies leads to beneficial results in many types of cancer, increasing the response rate compared to conventional therapies [77]. Also, IDO2 is a potential therapeutic target for diseases accompanied by immune escape, such as cancer. Compared to IDO1, its upregulation is more frequently observed in autoimmune reactions but is also found in cancer. The knowledge about IDO2 in the immune system is scarce, but evidence suggests that it supports IDO1 in mediating T regs suppressive mechanisms [78,79,80]. However, the detailed mechanisms of both isoforms in different diseases are still not fully understood.

In preclinical research 1-MT significantly reduced tumor-mediated immunosuppression, leading to the regression of systemic tumors and proving its utility in dealing with immune escape [37,38,39,40,81,82]. Additionally, its administration in combination with carboxyamidotriazole (CAI) potently increases the activity of cytotoxic CD8+ T cells and their ability to kill tumor cells, probably due to the reduced expression of programmed cell death protein-1 (PD-1) and increased IFN-γ secretion in the tumor microenvironment associated with decreased KYN level [83]. Additionally, inhibition of KYN synthesis due to blocking of IDO by 1-MT leads to an increase in T cell and NK cell proliferation [84,85,86,87]. Commonly used in preclinical research 1-MT is a racemic mixture of the two isoforms l-methyl-d-tryptophan and 1-methyl-l-tryptophan (D-1-MT). 1-MT as a racemic mixture mainly inhibits the IDO1 activity. Interestingly, the individual stereoisomers of 1-MT differ in their affinity for the particular isoforms of this enzyme. Authors discovered that the L stereoisomer of 1-MT is more potent in the inhibition of the IDO1 isoform. In turn, D-1-MT is less active in inhibiting IDO1 while it has a much greater affinity for IDO2 [44,88]. Preclinical studies also demonstrated that D-1-MT has superior antitumor activity and is more effective in inhibiting the activity of IDO-expressing tolerogenic DCs, than its L-stereoisomer [88,89]. In effect, d-1-MT, under the name indoximod, is developed clinically as the IDO2 inhibitor for the treatment of several cancers with the general aim of reversing cancer-associated immunosuppression [90,91,92]. Similar results were also observed when CAI was combined with 3′,4′-dimethoxyflavone (DMF), an AhR antagonist. The anticancer properties of CAI combined with DMF or 1-MT are higher than CAI in monotherapy and comparable to an anti-PD-1 antibody. These combinations seem to be an effective strategy for the treatment of various tumors [83]. Preclinical research showed that the combination of melatonin and 1-MT has a synergistic effect combined with tumor-specific immunotherapy and seems to be a new and promising method of growth control of HPV-associated tumors [93]. Also, novel IDO inhibitors, LW106 and NTRC 3883-0 inhibit tumor outgrowth in-vivo, limiting IDO-dependent stroma immune resistance and proliferation of cancer stem cells within the tumor microenvironment (Table 1) [94,95].

#### 3.1.1. Lung Cancer

**Figure 3:**
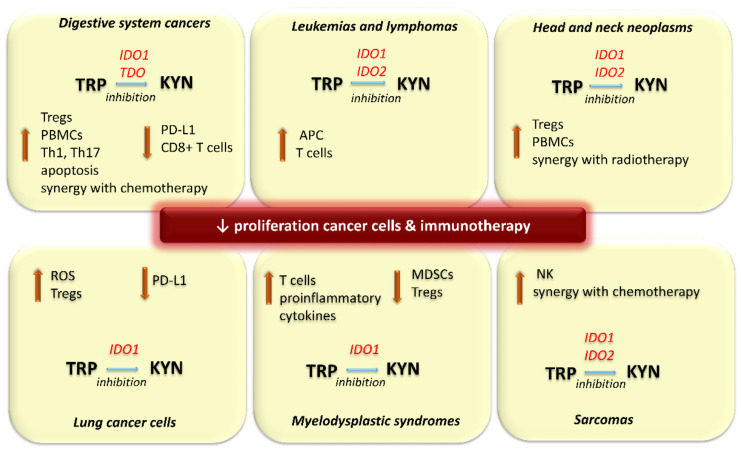
Effects of inhibition of 2,3-dioxygenases activity in selected types of cancers. APC: antigen presenting cells, CD8+ T cells: cytotoxic T cells IDO: indoleamine 2,3-dioxygenase, KYN: kynurenine, MDSCs: myeloid-derived suppressor cells, NK: natural killer cells, PBMCs: peripheral blood mononuclear cells, PD-L1: programmed cell death protein-1 ligand, ROS: reactive oxygen species, TDO: tryptophan 2,3-dioxygenase, Th: Helper T cells, TRP: tryptophan, Tregs: T regulatory cell.

Preclinical studies showed that anti-PD1 resistance is associated with IDO1 overactivity. PD-L1 expression correlates with IDO1. Therefore, it may be a novel target for lung cancer immunotherapy [110,111]. All current immunotherapies are accompanied by an activation of IDO, which is a negative feedback mechanism. This suggested to the researchers, that IDO inhibitors could potentially be used in combination with immune checkpoint inhibitors in the treatment of the anti-PD1-resistant tumors [112]. So far, these compounds seem to be the most promising solution of tumor resistance to immune checkpoint inhibitors. Li et al. discovered that the treatment with an IDO1 inhibitor navoximod restored the antitumor immune responses in anti-PD1 resistance conditions (Table 1). Therefore, the combination therapy with the inhibitor of IDO1 and the agent targeting PD-1/PD ligand 1 (PD-L1) was expected to be an effective option for overcoming the immunosuppressive tumor microenvironment in anti-PD1-resistant tumors, such as melanoma, glioblastoma multiforme (GBM), or non-small cell lung cancer (Figure 3) [113,146]. However, a phase I clinical trial of navoximod with a PD-L1 inhibitor (atezolizumab) in advanced solid tumors showed that although a pharmacologic effect of navoximod was observed, there was no clear evidence of clinical benefit from concomitant administration of navoximod with atezolizumab [147,148]. 

Cisplatin resistance (CR) is a significant problem in lung cancer therapy. CR lung cancer cells consume amino acids, such as glutamine and TRP for survival, rather than glucose. Additionally, CR cells can activate KP to reduce the excess of reactive oxygen species (ROS) and maintain conditions suitable for growth and proliferation. Knocking down IDO1 expression using short hairpin RNA or its pharmacological inhibition by epacadostat significantly increases ROS level and inhibits the growth of CR cells (Table 1). Exposing CR cells to antioxidants also leads to the suppression of IDO1 activity, indicating an interrelationship between ROS and IDO1. Moreover, the expression and activation of T regs in the tissues of CR tumors were higher, compared with cisplatin-sensitive tumors. Therefore, the use of KP enzyme inhibitors may be an effective method against CR in tumors [114].

#### 3.1.2. Breast Cancer

The IDO inhibitors ethyl pyruvate and amidoxime slightly suppress the growth of the Ehrlich tumor in mice and potentiates the therapeutic effect of cyclophosphamide [97]. In turn, EOS200271, the novel, selective, non-competitive, orally bioavailable IDO1 inhibitor, demonstrated a potent antitumor effect in the breast cancer animal model. It potently reduces intracellular KYN levels and IDO1-induced T cell anergy, and slows tumor cell growth, both in-vitro and in-vivo, in monotherapy and combined with antibodies directed against PD-L1 [131]. Furthermore, in an in-vitro model of triple-negative breast cancer, TDO2 inhibition by 680C91 significantly reduced intracellular KYN levels, leading to their increased sensitivity to the process of programmed cell death, as well as reduced their ability to proliferate, migrate and invade (Table 1) [71].

Cyclooxygenase-2 (COX-2), the enzyme responsible for prostaglandin synthesis, is upregulated in several tumors. Chen et al. demonstrated the anticancer effect of COX-2 inhibitor nimesulide in COX-2-overexpressing breast cancer in-vivo. They also proved its ability to inhibit IDO activity [123]. Simultaneous inhibition of IDO and COX-2 potently suppresses tumor growth (Table 1) [138,139]. Nimesulide significantly reduced systemic KYN synthesis and therefore could potentially target leukemia-induced IDO immune dysfunction as well [138]. Of course, it cannot be ruled out that, as with celecoxib, the reduction in IDO activity and inhibition of tumor growth may result from COX-2 inhibition, but this points to the potential utility of other COX-2 inhibitors to control IDO activation in cancer treatment [138,139,149].

#### 3.1.3. Leukemias and Lymphomas

Another novel IDO inhibitor, Toho-1, has been investigated for its potential use in cancer immunotherapy. In addition to solid tumors, the increase in the IDO expression induced by IFN-γ or lipopolysaccharide (LPS) is also observed in the PMDC05 leukemic plasmacytoid dendritic cell line. Treatment by Toho-1 enhanced the antigen-presenting and cytotoxic T cells’ activity against PMDC05 cells. It suggests its potential applicability in leukemia immunotherapy (Figure 3) [134]. As mentioned before, indoximod is a D-1-methylated TRP molecule with immune checkpoint inhibitory activity. It is a selective IDO2 inhibitor developed for antitumor therapies (Table 1). In combination with cyclophosphamide, it is effective for IDO-positive non-Hodgkin lymphoma treatment in the mouse model [137].

#### 3.1.4. Head and Neck Neoplasms

In addition to the above, the co-treatment with 1-MT and temozolomide or cytoxan prolonged survival rate in the intracranial GL261 glioblastoma multiforme (GBM) mouse model. It also increases the effectiveness of anticancer drugs against the glioma cell lines without additional side effects [104,150] Furthermore, IDO1 inhibition by 1-MT synergizes with radiotherapy in animal models of glioblastoma and Lewis lung cancer. However, 1-MT poorly penetrates the blood-brain barrier (BBB) [34,82,151]. Therefore, its effectiveness against glioma after systemic administration appears to be limited. In turn, tryptamine (TRY) and *N*-dimethyltryptamine (DMT) are novel non-competitive inhibitors effective against both constitutively expressed and IFN-γ-induced IDO activity. This suggests that they have the potential to be developed for IDO1- and TDO-related cancer treatment. They effectively inhibit the A172 human glioma cell line growth. Besides, TRY and DMT increased the cytotoxic activity of peripheral blood mononuclear cells in co-culture assays [108]. The commonly used antiviral agent acyclovir also inhibits IDO, leading to a decrease in T regs function in the GBM model [105]. In turn, IDO inhibition by 1-MT or dinaciclib, identified as an indirect inhibitor of this enzyme, in GBM and head and neck squamous cell carcinomas revealed a synergistic effect co-administered with temozolomide (Figure 3) [85,88,150]. Conventional chemotherapeutics, similarly to immune checkpoint blockers commonly activate KP. Numerous reports proved that it reduces their effectiveness [106]. In turn, the administration of IDO inhibitors in combination therapy may increase their efficacy. Therefore, these agents are considered as a promising treatment strategy for lymphoma, melanoma, glioma, glioblastoma and head and neck squamous cell carcinomas (Table 1) [83,105,106,107,108,109,118,119,134,137,151]. Indoximod is currently in clinical trials in combination with either temozolomide or bevacizumab for patients with GBM resistant to initial therapy and few objective responses have been observed [90,91]. This is another evidence that impairing tumor immune resistance seems to be a useful approach in antitumor therapy and IDO inhibition is effective in restoring immune function in malignancies dependent on T regs stimulation, such as GBM. In turn, a phase I clinical trial of EOS200271 showed promising results. It has a tolerable safety profile and significantly inhibits endogenous KYN synthesis, indicating effectiveness and durable clinical benefit in patients with recurrent malignant glioma [120].

#### 3.1.5. Melanoma

Melanoma patients treated with IFN α-2b have increased IDO activity, which leads to an increase in T regs and PD-L1 cytotoxic T cells amount. Therefore, inhibition of IDO1 activity may be an effective solution to this problem [121,152]. In addition, Jia et al. demonstrated that 1-MT co-administered with pimozide revealed combined effectiveness against melanoma in the animal model. 1-MT intensifies the antitumor immunity of pimozide against melanoma via the regulation of tumor proliferation, migration, apoptosis and immune response. In effect, co-treatment with 1-MT significantly prolonged the survival rate of tumor-bearing mice compared to those receiving pimozide in monotherapy. It suggests that the use of these compounds has great potential as a novel treatment strategy for this type of tumor [122]. Similarly, high hopes are attached to navoximod. In the B16F10 mouse model, co-administered with pmel-1 T cells and gp100 peptide vaccination almost completely reduced relative tumor size. Also, DX-03-12, an imidazole-based compound with a phenylimidazole scaffold, seems to be a promising candidate for the treatment of melanoma. It is a potent, orally bioavailable IDO1 inhibitor with low cellular toxicity and high in-vivo antitumor efficacy. Its administration inhibits tumor growth in a B16-F10 melanoma mouse model [153]. Likewise, LW-106 demonstrated a potent antitumor activity based on a similar mechanism in the IDO-overexpressing melanoma mouse model, while NTRC 3883-0 in ovarian cancer cell culture and melanoma mouse model (Table 1) [94,95].

Epacadostat is another IDO inhibitor entered into clinical trials. It appeared to be a promising candidate for the treatment of melanoma, showing decent efficacy in inhibiting its growth in-vivo (Table 1) [94]. However, a phase I clinical trial with epacadostat and PD-L1 blockade in patients with advanced solid tumors did not bring positive results. This combination was not effective to be pursued in PD-L1 negative patients [154]. Despite these unsatisfactory results, this compound is still undergoing further clinical evaluations in combination with ipilimumab and pembrolizumab in melanoma and as monotherapy in advanced ovarian cancer. It recently underwent the phase I/II clinical trials and is currently during phase III trials in combination with checkpoint blockade. However, the latest results from one of the phase III clinical studies indicate that it does not improve the effectiveness of pembrolizumab in patients with unresectable or metastatic melanoma [74,155,156,157].

#### 3.1.6. Ovarian and Cervical Cancer

Preclinical research showed that IDO1 inhibition by 1-MT also counteracts the invasion of carboplatin-resistant ovarian cancer cells via the down-regulation of IDO expression and re-activation of immune cell function. Thus, inhibition of the KP activity seems to be a potentially effective solution in the case of carboplatin-resistant cancers (Table 1) [46,129]. 

β-Lapachone is a naturally occurring 1,2-naphthoquinone-based compound, that advanced into clinical trials, due to its tumor-selective cytotoxic properties. It turned out to be an uncompetitive IDO1 inhibitor, which potently decreases the proliferation rate of cervical cancer cells in-vitro (Table 1). Its antitumor effect seems to be also related to its impact on TDO activity. Flick et al. showed that the effect of β-lapachone on cancer cells is related to the synergism of its cytotoxic and immunoregulatory properties. This discovery expands its potential use as an anticancer agent [140]. 

The limited clinical activity of epacadostat prompted the researchers to develop new compounds able to decrease IDO-dependent immune dysregulation in cancer. LVS-019 is a recently discovered potent IDO1 inhibitor. However, the impact of this compound on tumor growth is not known yet. In turn, its derivative, LBJ-10, effectively inhibits the proliferation of cervical cancer cells in-vitro via IDO1 inhibition. Therefore it has a high potential for further development as the agent for anticancer therapy [141]. In turn, BMS-986242 is another novel IDO1 inhibitor that was originally designed to treat various cancers, including melanoma and renal cell carcinoma (RCC). Interestingly, recent in-vitro and animal model studies indicate its high efficacy and safety in the treatment of ovarian cancer (Table 1). Promising results from preclinical studies have led the compound to be included in Phase I/II clinical trials in combination with nivolumab in patients with advanced cancer [130].

#### 3.1.7. Digestive System Cancers

Both systemic and local administration of the IDO inhibitor, ethyl pyruvate accelerated tumor regression in the H-29 liver cancer-bearing mice [142]. In turn, 3-(2-(pyridyl)ethenyl) indole derivatives are new, potent and promising inhibitors of TDO. Their conjugates with irinotecan, designed to reverse tumor immune suppression, significantly improved the efficacy of irinotecan in HepG2 cancer cells. In-vitro studies showed that their use significantly increases the rate of apoptosis of cancer cells by inducing a mitochondria-dependent mechanism. They also potently downregulate the expression level of TDO. This, in turn, intensifies the inhibition of KYN synthesis, leading to intensified proliferation and activation of T cells (Figure 3). In-vivo studies proved that these conjugates can improve the tumor immune microenvironment in a murine model of hepatocellular carcinoma (Table 1). This combination of chemotherapy and immunotherapy may be a promising strategy for treating this type of cancer [143].

Pancreatic ductal adenocarcinoma (PDAC) belongs to tumors resistant to immune checkpoint inhibitors. Blaie et al. showed that a granulocyte-macrophage colony-stimulating factor-secreting allogeneic pancreatic tumor cell vaccine (GVAX) induces intratumoral T cell infiltration, leading to the activation of the tumor microenvironment. They also proved that GVAX therapy induces IDO1 expression within tumor epithelia and vaccine-induced tertiary lymphoid aggregates [132]. In addition, in PDAC, IDO1 expression plays an important role in regulating the activity of TH1, TH17 and probably T regs [131,132] (Figure 3). Epacadostat and EOS200271, a novel IDO1 inhibitor, enhance the antitumor efficacy of GVAX in the PDACs murine model, intensifying intratumoral T cell infiltration and activity caused by the vaccine. Furthermore, an administration of EOS200271 during the vaccine therapy diminished the suppressive effect of intratumoral myeloid-derived suppressor cells (MDSCs) on the proliferation of CD8+ T cells (Table 1). However, the addition of the anti–PD-L1 antibody to this combination did not cause further synergy and in fact may have a negative impact, decreasing the number of intratumoral effector T cells [131,132,133]. It suggests that EOS200271 does not increase the effectiveness of anti–PD-1/PD-L1 antibody for T cell–inflamed tumors such as PDACs treated with GVAX therapy.

Since IDO1-dependent KYN synthesis has been shown to directly promote colorectal cancer (CRC) growth through β-catenin activation, IDO inhibition has emerged as a promising strategy for CRC prevention. IDO overactivity is observed even in the early stages of colon carcinogenesis. Its inhibition suppresses the growth and stimulates the apoptosis of cancer cells [63,64,66,98,158,159,160,161,162,163]. Therefore, the use of IDO inhibitors, such as 1-MT, or epigallocatechin gallate in the animal models suppressed the occurrence of colonic preneoplastic lesions and reduced tumor size and mortality in animals with cancer (Figure 3) [63,98,99,163]. Similar effects were also observed with newer IDO1 inhibitors, such as epacadostat, navoximod and amidoxime. It confirms the potential utility of IDO1 inhibitors as agents for the chemoprevention of IDO-expressing colonic neoplasms. Furthermore, the regulation of IDO1 activity may be considered as a target for the prevention of other malignancies [98,99,100,101,155,163,164]. 

Also, 4,6-disubstituted indazole derivatives demonstrated the potential utility as IDO1 inhibitors in cancer treatment. In turn, 4,6-substituted-1H-indazole derivatives and 4-bromophenylhydrazinyl benzenesulfonylphenylureas showed high activity against both IDO and TDO and inhibited cancer growth and reduce the tumor weight in-vivo in the CT26 xenograft model (Table 1) [96,100,102]. This suggests that they have the potential to be developed for IDO1- and TDO-related cancer treatment.

#### 3.1.8. Renal Cancer 

Changes in TRP metabolism are also observed in the course RCC and lead to the increased synthesis of immunosuppressive metabolites. The inhibition of KP activity in the presence of IFN-α decreases RCC growth in the murine model. The IDO1 inhibitor methyl-thiohydantoin-DL-TRP alters the functionality of intratumoral leukocytes, improving the therapeutic effects of IFN-α (Table 1). Therefore, the combination of IFN-α with IDO inhibitors should be assessed for its utility in the treatment of RCC [135].

#### 3.1.9. Myelodysplastic Syndromes

Recent studies also indicate the role of IDO-dependent immune dysregulation in the pathogenesis of myelodysplastic syndromes (MDS). The expansion of inflammatory hematopoietic suppressor cells, called MDSCs, impairs hematopoiesis and leads to the development of this disease. In turn, increased proliferation and activity of T regs are associated with worse disease outcomes [165,166,167,168,169]. Epacadostat is an oral, potent and selective IDO1 inhibitor. It does not affect IDO2 or TDO activity. Preclinical data suggest that its administration increases proliferation and activity of cytotoxic T cells and decreases T regs and MDSCs. It also promotes proliferation, survival and functions of various immune and immunoregulatory cells. These pleiotropic effects significantly intensify host antitumor immunity in-vivo. Simultaneous treatment with epacadostat, IFN-γ and LPS did not change the phenotype of matured human DCs and decreased KYN production. Peptide-specific T cell lines stimulated with DCs treated with epacadostat produced significantly more proinflammatory cytokines and show higher tumor cell lysis ability (Figure 3). These results indicate that epacadostat could increase the effectiveness of immunotherapeutics, including cancer vaccines, to increase inflammatory responses (Table 1) [124,170]. 

#### 3.1.10. Sarcomas

Sarcomas revealed limited vulnerability to immunotherapy. In the preclinical studies, PD-L1 blockade led to the increase in the activity of KP enzymes, including both IDO1 and IDO2 [170,171]. IDO1 inhibition by navoximod resulted in a significant decrease in KYN level and KYN/TRP ratio in tumor cells and plasma. However, this compound did not show direct antitumoral activity. Regardless, it induced down-regulation of poliovirus receptor and granzyme expression, as well as up-regulation of inhibin and Dtx4 ubiquitin ligase, suggesting a potential effect of KP metabolites on NK cell function. It indicates that IDO1 inhibition stimulates NK cells against tumor cells (Figure 3). Besides, the co-administration of navoximod with paclitaxel displayed synergistic antitumor effects and did not induce additional side effects. It demonstrates that the combination of IDO inhibitor-based immunotherapy with chemotherapy has great potential as the new strategy for sarcomas treatment (Table 1) [136]. 

### 3.2. The Role of Intracellular Mechanisms of Kynurenine Pathway Modulation in Tumor Therapy

Suberoylanilide hydroxamic acid and bortezomib (BTZ) downregulate the IFN-γ-induced IDO expression via inhibition of the phosphorylation and nuclear translocation of STAT1, which causes a suppression of the IDO transcription [126,127]. Similarly, sodium butyrate (NaB) decreases IDO expression, through a mechanism involving increased STAT-1 acetylation. All these compounds reduce IDO-mediated immune tolerance [128]. This suggests that the Janus kinase/signal transducers and activators of transcription (JAK/STAT1) signaling pathway plays an important role in regulating IDO expression. Furthermore, BTZ demonstrated antitumor activity against nasopharyngeal carcinoma (NPC) cells, while in-vitro and in combination with the suberoylanilide hydroxamic potently induces apoptosis and suppresses the growth of NPC in mouse model [127,128]. Therefore, BTZ in combination with NPC-targeted tumor vaccines seems to be useful in the prevention and treatment of this type of cancer (Table 1). High activity of the JAK/STAT1 pathway is also observed, among others, in gallbladder cancer cells. Therefore, its pharmacological modulation may also be a promising method to break tumor immune tolerance and improve immunotherapies of other cancers [123,126,128]. 

Fludarabine, another inhibitor of STAT1 phosphorylation, also revealed effectiveness in the suppression of IDO activity in breast cancer and melanoma cell lines. However, its mechanism different is from other inhibitors. It is associated with the modulation of STAT1 activation. It does not affect directly the level of IDO transcription but participates in IDO post-transcriptional control. Fludarabine enhances IDO degradation through a proteasome-dependent pathway. Currently used in the treatment of certain malignancies, it has also demonstrated the ability to promote the T cell-mediated antitumor response by inducing degradation of the IDO protein. As it impairs T cell-dependent IDO upregulation, its use in the pre-treatment before immunotherapies, involving the response of T cells, may potentially prevent upregulation of IDO-dependent immunosuppressive mechanisms (Table 1). Also, combining fludarabine with other IDO inhibitors or immune-checkpoint modulators cal significantly reduces immunosuppression accompanying immunotherapies [123]. 

The therapeutic effect of imatinib, a tyrosine kinase inhibitor, in gastrointestinal stromal tumors (GIST) is also associated with downregulation of IDO1 expression, which, in turn, is driven by the oncogenic tyrosine-protein kinase KIT signaling. Imatinib in experimental tumor models potently reversed IDO1-mediated immunosuppression. This finding suggests that targeting IDO1 may provide a benefit in the context of imatinib treatment of GIST. The associations between cytotoxic T-lymphocyte-associated protein 4 (CTLA-4) and IDO expression indicate, that the combination of the KIT blockade with an anti-CTLA-4 therapy seems to be an effective approach in the management GIST (Table 1) [103]. 

Luo et al. examined the impact of a polysaccharide derived from the Lepista sordid (LSP) on IDO activity in HepG2 cells. LSP treatment potently inhibits IDO-dependent KYN synthesis in IFN-γ-treated HepG2 cells [144]. Furthermore, resveratrol, a stilbenoid, a type of natural phenol, also inhibits the expression and activity of IFN-γ-induced IDO in bone marrow-derived dendritic cells (BMDCs). The mechanism of these compounds seems to be associated with the inhibition of the JAK/STAT1 pathway and protein kinase Cd. Furthermore, in the case of resveratrol, IDO suppression in IFN-γ-stimulated BMDCs plays a key role in the antitumor response, because it modulates the activity of cytotoxic T cells. Systemic administration of resveratrol also suppressed tumor growth in EG7 thymoma-bearing mice in the IDO-dependent manner [117]. It demonstrates that resveratrol modulates the immune response and the IDO-mediated immune tolerance. However, resveratrol not only exerts the antitumor effect by the modulation of immune cells’ activation but also may enhance IDO-mediated tolerance within the tumor environment and tumor cells. Also, Feiji Recipe, a Chinese herbal medicine compound, inhibits tumor growth and prolongs survival in patients with lung cancer. In the mouse model of this cancer, it effectively decreases T cell apoptosis and proliferation of T regs by reducing IDO expression (Table 1). However, its exact mechanism remains unknown [115].

Interferon-induced guanylate-binding protein 1 (GBP1)-mediated extracellular secretion of IDO1 stimulates the progression of lung cancer cells. The interaction between GBP1 and IDO1 proteins additionally enhances the extracellular secretion of this enzyme. Meng et al. demonstrated that inhibition of this interaction by stragaloside IV or astragaloside IV causes a decrease in the extracellular secretion of IDO1 and increases the antitumor effect of PD-1 inhibitors in lung cancer cells both in-vivo and in-vitro (Table 1) [116].

Carbidopa is commonly used to treat Parkinson’s disease (PD) as a peripheral levodopa decarboxylase inhibitor. Patients administering this drug have a significantly lower incidence of various tumors. Recently, it has been shown that it also inhibits the activity of TDO and KYNU within the tumor tissue and its growth. However, it was not effective in inhibiting the proliferation of breast cancer and melanoma cells. It appears to be associated with the increase in an indole-3-acetonitrile level during carbidopa treatment, which enhances the viability of these types of cancer cells. It suggests that treatment with carbidopa may alter TRP metabolism in breast cancer and melanoma in a way that promotes the formation of pro-proliferative TRP metabolites, which may contribute to their development [172]. 

### 3.3. Angiogenesis Inhibition

Tumor angiogenesis is a key process necessary for the growth, metastasis and transfer of essential nutrients for solid tumors. Its inhibition is recognized as an effective anticancer strategy for non-small-cell lung carcinoma [173]. IDO revealed non-immune function, in the regulation of this process [174]. Erianin, a natural product isolated from *Dendrobium chrysotoxum* Lindl, downregulates the IDO-induced tumor cells angiogenesis and endothelial cell-dependent angiogenesis by targeting the JAK2/STAT3 pathway and its downstream genes, such as matrix metalloproteinases-2 and -9 (Table 1). Also, administration of other IDO inhibitors may be effective in inhibiting the IDO-dependent angiogenesis in lung cancer, as well as in other malignancies, providing a potential solution in preventing the processes of tumor growth and metastasis [175,176].

*Astragalus membranaceus*, commonly used in traditional Chinese medicine, possesses anti-inflammatory and antitumor properties. Several studies indicate that its extract (PG2) significantly inhibits the growth of tumor cells. Connexin 43 (Cx43) is a component of gap junctions, which allow for gap junction intercellular communication between cells. It is omnipresent in cells and involved in the passage of chemotherapeutics into the tumor and its environment cells. The treatment of PG2 upregulates the expression of Cx43, enhancing the distribution of chemotherapeutics, but also downregulates IDO expression. The PG2 and cisplatin co-treatment in animal models of melanoma and lung cancer significantly slowed tumor growth and prolonged survival. This indicates the potential value of the combination therapy of PG2 with chemotherapeutics [177].

### 3.4. Pharmacological Modulation Activity of KMO Activity

Chauhan et al. discovered that dysfunctional plasmacytoid DCs contribute to the pathogenesis of multiple myeloma (MM). Interaction between plasmacytoid DCs and MM cells triggers upregulation of KMO expression in MM cells [178]. Pharmacological inhibition of this enzyme using Ro-61–8048 activates plasmacytoid DCs and enhances their ability to trigger autologous proliferation of T cells. In effect, the administration of Ro-61–8048 leads to an increase in T cells cytotoxic and NK cells cytolytic activity against tumor cells (Table 1). Furthermore, the combination of Ro-61–8048 and immune checkpoint PD-L1 blockade potently enhances antitumor immunity and cytotoxicity against MM cells, more potent than either agent alone. These preclinical results provide the basis for a novel immune-based therapeutic strategy, targeting KMO, alone or in combination with PD-L1 blockade, to restore anti-MM immune responses in patients with this malignancy [145].

### 3.5. Gene Silencing

Genetic manipulation is another promising option for targeting TRP metabolism in cancer. Silencing of the IDO1 gene, using RNA interference, significantly reduced melanoma growth in-vivo. Moreover, it also significantly increased the efficacy of DC-based vaccines. Importantly, this approach can target specific cell types and can also inhibit other KP enzymes, even in parallel. Furthermore, gene silencing also eliminates any non-enzymatic tolerance promoting the IDO1 protein activity [179,180]. Another highly effective strategy to block KP activity is to inhibit IDO1 and TDO2 expression using locked nucleic acid (LNA)-modified antisense oligonucleotide. They efficiently downregulate the expression of these enzymes in cancer cells in-vitro without using transfection reagents. Treatment of cancer cells in-vitro with IDO1-specific and/or TDO2 ASOs and small molecule inhibitors can reduce the production of KYN by cancer cells in a synergistic manner and increase proliferation of activated T cells in the co-culture [181]. Therefore, a combination of gene silencing using RNA interference or LNA-modified ASOs and small molecule inhibitors should be considered as a strategy for managing KYN-dependent immunosuppression in cancer immunotherapy.

### 3.6. Blockade of the AhR

As mentioned before, KYN activates the AhR, inducing target genes of this receptor, including the AhR–IL-6–STAT3 pathway. Due to increased production of KYN, the AhR and, in effect, all signaling pathways dependent on this receptor are overactivated in IDO- and TDO-overexpressing tumors. This, in turn, enhances mechanisms responsible for the development of resistance against immune checkpoint inhibitors and tumor immune escape [18,19,20,71,83,182,183]. AhR-mediated immunosuppression can be reversed by direct blockade of this receptor [18,19,20,71,83,182]. The administration of selective AhR antagonists, such as KYN-101, or the previously mentioned CH-223191 and DMF significantly slows the progression of IDO- and TDO-overexpressing tumors in-vivo [71,83,114,182,183]. Besides, these agents synergize with PD-1 blockade during co-treatment. Campesato et al. also suggested that direct blocking the AhR activation, using its antagonists, in IDO- and TDO- overexpressing tumors could overcome the limitation of single IDO or TDO targeting agents, such as enzyme inhibitors and has the potential as a novel approach to immunotherapy, especially combined with the immune checkpoint inhibitors [182].

## 4. Summary and Future Directions

IDO inhibitors, especially targeting IDO1 isoform, are an important class of pharmaceuticals because KYN and its metabolites directly participate in many physiological and pathological processes, including tumor immune escape, development of infections, autoimmune, or metabolic diseases and neurodegeneration [2,3,4,5,6,7,8,9,10,11,12,13,14,15,16,17,18,19,20,21,22,37,38,39,40,41,42,43,44,45,46,47,72,77,81,100,184]. They are mostly considered as therapeutics useful in cancer therapy [72,77,82,100,101,125,184,185,186]. Although IDO1 targeting in monotherapy has been shown to have disappointing efficacy in preclinical cancer models, their combinations with conventional treatments, immunogenic chemotherapy, or immunosuppressive drugs have shown satisfactory results. Moreover, the mechanism and safety profile of IDO1 inhibitors are well suited to their use in combination therapies. This indicates that drugs targeting KP should be combined with other anticancer therapies, to improve their efficacy [74,77,81,101,164,185,186,187]. To date, only a few oral IDO inhibitors have entered clinical trials, including epacadostat, indoximod, navoximod, BMS-986242 and EOS200271. In contrast, newer compounds capable of inducing an effective reduction in intracellular KYN levels remain in preclinical studies [76,82,90,91,121,125,130,147,148,152,184,186,187,188,189]. 

It is worth mentioning a few controversies regarding the use of IDO inhibitors. Competitive inhibitors, such as 1-MT, widely used in preclinical studies, or norharmane, hardly induce therapeutic effect in-vivo due to their low potency. They reach effectiveness only at a high, comparable with TRP, plasma level [77,81,88,164,189,190,191]. Besides, they fulfill many of the structural and functional criteria as the potential AhR agonists with a binding affinity similar to KYN. Therefore, their low systemic immunomodulatory effect seems to be also to a certain degree related to their off-target effects, such as the activation of AhR [146,192]. This should be taken into account when evaluating their clinical outcomes and potential utility. In contrast, newer, more potent inhibitors such as epacadostat, navoximod, or BMS-986242 effectively inhibit IDO activity even on nanomolar concentrations, efficiently decreasing KYN production in-vivo. However, their direct antitumor effect in monotherapy remains low [88,154,157,164,193,194,195,196]. This all indicates that a better understanding of the basic biology of the KP enzymes is still needed to develop new, more effective and safer drugs and identify patients who are best suited to benefit from them. Despite these limitations and the failures observed in clinical trials of epacadostat, combination therapies with IDO1 inhibitors and other immuno- or chemotherapeutics for the treatment of cancer still appear to be clinically justified because IDO1 is commonly overactivated in tumors and their microenvironment, both constitutively and induced by the antitumor immune response. [43,44,45,46,47,181]. Additionally, recent preclinical evidence indicates that the IDO1 inhibitors synergize the effects of tumor immuno- and chemotherapies and help to solve the problem of tumor resistance against them [83,93,105,106,107,108,112,113,119,120,129,132,133,136,137,143,146,151,154,155,156,157,171]. In turn, the discoveries concerning the role of IDO1 in tumor angiogenesis suggest that inhibiting its activity may effectively suppress this process, providing a potentially effective solution in the prevention of tumor development and metastasis. However, the impact of IDO inhibitors on tumor vasculature requires better examination [174,175,176].

Beyond the direct inhibition of IDO1, the authors suggest other approaches, which may serve as potential therapeutic targets associated with impaired TRP catabolism. Inhibition of TDO and IDO2 may limit KYN synthesis when inhibition of IDO1 is not enough to achieve satisfactory clinical effects [78,89,100,136,143]. The direct pharmacological targeting of TDO is considered for cancer immunotherapy [73,74,75,96,100,140,143]. Also, the development of dual IDO and TDO inhibitors should extend the therapeutic possibilities and improve the effectiveness of cancer treatment. Targeting more than one enzyme at once may induce broad and synergistic responses of the immune system. It appears to be an effective solution in the case of inherent or acquired resistance to currently available IDO1 inhibitors [76,77,100]. Because tumor cells can upregulate multiple immune checkpoint pathways to evade the immune response, therefore, selective inhibition of IDO1 may not be sufficient to promote tumor regression in most patients. The idea of dual IDO1 and TDO inhibitors is also desirable, because inhibition of IDO may induce the activation of TDO [96,100,164]. Moreover, targeting IDO2 together with IDO1 may open new options for cancer immunotherapy [78,81,101,136]. However, they are upstream enzymes of KP and their inhibition may cause more adverse effects than selective more selective approaches [78,100,164].

Pharmacological inhibition of KMO also seems to have great potential as the therapeutic target for the treatment of certain neoplasms, such as MM [145]. Furthermore, some authors observed that the expression levels of KMO and KYNU are potently upregulated in the course of HER2-enriched and triple-negative breast cancer subtypes, as well as cutaneous squamous cell carcinoma. It leads to excessive production of 3-HAA and AA, other potent immunosuppressive metabolites of KP. Their accumulation intensifies the progression of these cancers. KMO and KYNU inhibitors may therefore be considered as potential options for the treatment of breast cancer and cutaneous squamous cell carcinoma [197,198]. However, the efficacy of these compounds in cancer treatment has not yet been evaluated.

In addition to direct inhibition of the enzyme, the authors outlined other targets that may be considered as potential therapeutic solutions for KP overactivation accompanying cancer progression. They include downregulation of IDO1, IDO2, TDO and KMO expression, upregulation of their proteasomal degradation rate, targeting upstream regulators of IDO1 expression and activation, such as JAK/STAT3 pathway and KIT tyrosine kinase or downstream effectors of the KP metabolites, such as general control nonderepressible-2 kinase and mammalian target of rapamycin kinase [40,103,115,116,123,126,127,128,144]. Furthermore, inhibition of extracellular IDO secretion or the direct targeting of particular KP metabolites using monoclonal antibodies as well as blocking the KP receptors such as AhR and G protein-coupled receptor 35, or TRP cellular transport mechanisms also should be taken into consideration for further therapeutic strategies [40,116]. These mechanisms seem to be the most promising, because they may reduce the immunosuppressive tumor microenvironment conditions, regardless of the source and intensity of KYN synthesis. Especially AhR antagonists, such as DMF, CH-223191 and KYN-101 showed promising results in antitumor therapy in animal models [71,83,114,182].

## 5. Conclusions

Numerous studies indicate that activation of the KP is observed during the course of various malignancies. In turn, the accumulation of metabolites belonging to this pathway may enhance neoplastic progression, because they stimulate tumor growth and inhibit the immune system’s response against cancer cells. They also appear to be at least partially responsible for the development of the resistance to antitumor therapy. It indicates that the modulation of the KP activity can be an effective solution as an adjunctive treatment in the management of numerous neoplasms. Among the enzymes belonging to this pathway, IDO has attracted the greatest interest among researchers Inhibition of its activity has so far provided the most promising results, both in-vitro and in-vivo. Also, TDO and KMO appear to have potential as possible targets for antitumor therapy. Despite the unpromising results of previous clinical trials with IDO inhibitors, numerous preclinical studies suggest that the inhibition of the KP enzymes may still prove to be an effective solution in cases remaining a challenge for modern oncology, especially the tumor immune escape and resistance to chemotherapy and immunotherapy. This demonstrates the rationale for further research in this area, especially for their clinical applications.

## Figures and Tables

**Table 1:** Targeting KP enzymes in preclinical studies on antineoplastic therapy.

Tumor	Target	Mechanism of Action and Agent	Suppression of Tumor Growth	Comments	References
**Colon cancer**	IDO1	**Direct enzyme inhibition**		**In-vitro** SW837 cell line culture **In-vivo** Balb/c mice bearing CT26 tumor	[64,96,97,98,99,100,101,102]
		1-MT	7%		
		4,6-substituted-1H-indazole derivatives	38%		
		Amidoxime	N/A		
		Epacadostat	56%		
		epigallocatechin gallate, Navoximod	N/A 75% (in co-treatment with oxaliplatin)		
		4-Bromophenylhydrazinyl benzenesulfonylphenylureas	25%		
**Ehrlich ascites carcinoma**	IDO1	**Direct enzyme inhibition**		**In-vivo** ICR mice bearing Erlich tumor	[97]
		Ethyl pyruvate	78% (in co-treatment with cyclophosphamide); 79% (in co-treatment with cycloplatam)		
		Amidoxime	88% (in co-treatment with cyclophosphamid); 85% (in co-treatment with cycloplatam)		
**Gastrointestinal stromal tumors**	IDO1	**Downregulation of oncogenic KIT tyrosine-protein kinase signaling**		**In-vivo** C57Bl/6J mice bearing GIST-T1 tumor	[103]
		Imatinib	70%		
**Glioblastoma**	IDO1	**Direct enzyme inhibition**		**In-vivo** C57BL/6 mice bearing GL261 glioblastoma	[83,104,105,106,107]
		1-MT	63% (in conjunction with two-fraction radiotherapy)		
		Dinaciclib	N/A		
		Acyclovir	N/A		
**Glioma**	IDO1	**Direct enzyme inhibition**		**In-vitro** A172 human glioma cell line culture **In-vivo** C57BL/6 mice bearing LN229 glioma	[107,108,109]
		1-MT	87% (in co-treatment with temozonlomide)		
		Tryptamine	N/A		
		*N*-dimethyltryptamine	64% (in combiantion with carboxyamidotriazole and cytotoxic T lymphocytes therapy)		
**HPV-associated tumors**	IDO1	**Direct enzyme inhibition**		**In-vivo** 57BL/6 mice bearing TC-1 tumor	[93]
		1-MT	60% (in combiantion with gDE7-based immunotherapy)		
**Lung cancer**	IDO1	**Direct enzyme inhibition**		**In-vitro** NCI-H460 and A549 cell line cultures **In-vivo** C57BL6/N mice bearing LLC, LL24 tumor lines, Balb/c nude mice bearing NCI-H1299 tumor	[94,95,110,111,112,113,114,115,116]
		1-MT	47%		
		Navoximod	65%		
		EOS200271	45%		
		LW106	68%		
		Epacadostat	51%		
		INCB023843	22%		
		**Downregualtion of enzyme expression**			
		Feiji Recipe	60%		
		**Inhibition of the extracellular IDO1 secretion**			
		Stragaloside IV	N/A		
		Astragaloside IV	72% (in combination with anti-PD1 antibody)		
**Thymona**		**Downregualtion of enzyme expression**		**In-vivo** C57BL/6 mice bearnng EG7 tumor line	[117]
		Resveratrol	51%		
**Melanoma**	IDO1	**Direct enzyme inhibition**		**In-vitro** 624.38mel cell line culture **In-vivo** BALB/c mice bearing B16F10 melanoma	[94,95,113,118,119,120,121,122,123]
		1-MT	52% (in co-treatment with cyclophosphamide) 48% (in co-treatment with pimozide) 46% (in monotherapy),		
		Navoximod	95% (in co-treatment with pmel-1 T cells and gp100 peptide vaccination)		
		Epacadostat	50%		
		DX-03-12	72.2%		
		LW106	65%		
		NTRC 3883-0	20%		
		**Increased proteasomal degradation**			
		Fludarabine	N/A		
**Myelodysplastic syndrome**	IDO1	**Direct enzyme inhibition**		**In-vivo** C57BL/6N mice bearing MDS92 tumor	[124,125]
		Epacadostat	50%		
**Nasolaryngeal carcinoma**	IDO1	**STAT1 acetylation**		**In-vitro** CNE2 and CNE1 cell line cultures **In-vivo** BALB/C (NU/NU) mice bearing C666-1 tumor	[126,127,128]
		Bortezomib	23%		
		Suberoylanilide hydroxamic acid	41%		
		Combination of bortezomib with suberoylanilide hydroxamic acid	76%		
		Sodium butyrate	N/A		
**Ovarian cancer**	IDO1	**Direct enzyme inhibition**		**In-vitro** SKOV3 cell line, patient-derived ovarian cancer cell culture **In-vivo** Balb/c NU/NU mice bearing SKOV3 tumor	[46,94,95,129,130]
		1-MT	10%		
		NTRC 3883-0	N/A		
		LW106	N/A		
		BMS-986242	N/A		
**Pancreatic Ductal Adenocarcinoma**	IDO1	**Direct enzyme inhibition**		**In-vitro** Human PDAC specimens	[124,131,132,133]
		Epacadostat	55%		
		EOS200271	32%		
**Plasmacytoid dendritic cell leukemia**	IDO1	**Direct enzyme inhibition**		**In-vitro** PMDC05 cell line culture	[134]
		Toho-1	N/A		
**Renal Cancer**	IDO1	**Direct enzyme inhibition**		**In-vivo** Balb/cJ mice bearing 786-O tumor	[135]
		Methyl-thiohydantoin-DL-TRP	45% (in co-treatment with IFN-α)		
**Sarcomas**	IDO1	**Direct enzyme inhibition**		**In-vivo** C57BL/6N mice bearing MCA205 tumor	[136]
		Navoximod	0% (did not show antitumoral activity, but has a potential impact on the NK cell functions).		
**Non-Hodgkin Lymphoma**	IDO2	**Direct enzyme inhibition** Indoximod	77% (in co-treatment with cyclophosphamide)	**In-vivo** BalB/c NU/NU mice bearing A20 B-cell lymphoma	[137]
**Breast cancer**	IDO 1	**Direct enzyme inhibition**		**In-vitro** MDA-231and BT549 cell lines **In-vivo** C57BL/6 mice bearing MDA-231 tumor	[71,123,131,138,139]
		EOS200271	21%		
		**Downregulation of enzyme expression**			
		Nimesulide	54%		
		Celecoxib	52%		
		**Increased proteasomal degradation**			
		Fludarabine	N/A		
	TDO2	**Direct enzyme inhibition**			
		680C91	85%		
**Cervical cancer**	IDO1	**Direct enzyme inhibition**		**In-vitro** HeLa cell line	[140,141]
		LBJ-10	N/A		
	IDO1 and TDO	**Direct enzyme inhibition**			
		β-lapachone	N/A		
**Liver cancer**	IDO1	**Direct enzyme inhibition**		**In-vitro** HepG2 cell line **In-vivo** BALB/c mice bearing H22 tumor C3HA mice bearing H-29 tumor	[142,143,144]
		Ethyl pyruvate	39%		
		**JAK/STAT1 pathway inhibition**			
		Polysaccharide from Lepista sordid	N/A		
	TDO	**Direct enzyme inhibition**			
		3-(2-(Pyridyl)ethenyl) indole derivatives	65% (in combination with irinotecan)		
**Multiple Myeloma**	KMO	**Direct enzyme inhibition**		**In-vitro** Patient-derived multiple myeloma cell culture	[145]
		Ro-61–8048	N/A		

IFN-α—interferon-α, N/A—Data not available.

## Data Availability

Data sharing not applicable.
